# Numerical Simulation and Deep Neural Network Revealed Drag Reduction of Microstructured Three-Dimensional Square Cylinders at High Reynolds Numbers

**DOI:** 10.3389/fbioe.2022.885962

**Published:** 2022-06-29

**Authors:** Siying Wang, Qibiao Wu, Xiaotao Shi

**Affiliations:** ^1^ Department of Engineering Structure and Mechanics, School of Science, Wuhan University of Technology, Wuhan, China; ^2^ Hubei International Science and Technology Cooperation Base of Fish Passage, China Three Gorges University, Yichang, China

**Keywords:** square cylinder, numerical simulation, passive control, drag reduction, neural network

## Abstract

Square cylinders are widely used in various fields. For example, they are common structures in fishways. The flow around square cylinders has been a common problem in various fields. However, reducing the flow drag of the square cylinder is a problem that remains unexplored. Many previous studies have reported the drag reduction of 2D square cylinders, which failed to reflect the drag of real structures. Also, some studies focus on the drag force of the inner wall of the square cylinder modified by the microstructure. Achieving drag reduction by microstructuring the surface of the 3D square cylinder is a challenging problem. This study applied a 3D numerical simulation and deep neural network to study the drag reduction performance of the square cylinder under different patch sizes. We studied the drag reduction performance of protrusion and pit-patched square cylinders and tried to find the rule between drag reduction performance and patch configuration. The results show that the square cylinder has better drag reduction performance in some cases. However, its drag reduction performance is greatly affected by the protrusion structure. Also, too large protrusions will increase the drag force of the structure. When the surface protrusion accounts for 10% of the total area of the square cylinder, the drag reduction performance is the best (22.1%). The pit patch structure demonstrated an insignificant drag reduction performance and even increased the drag in most cases. The DNN prediction model demonstrated the robustness of the numerical simulation data.

## Introduction

Square cylinders are widely used in the engineering field, and they are common structures in fishways. A square cylinder can be regarded as a bluff body structure with a rectangular cross section, such as cylinders, beams, and fences in the building structure (Chen, 2019; Shui et al., 2021; Yang et al., 2021). In fluid mechanics, the flow around a square cylinder is a classic problem of flow around a bluff body, which contains complex phenomena such as impact, separation, reattachment, orbiting, and vortex often encountered in various fields (Lahaye et al., 2014; Vikram et al., 2020). In addition, many engineering structures and marine cylinder structures involve flow around square cylinders (Behera and Saha, 2021). The study on square cylinders generally focuses on the drag and lift forces and the local instability oscillation caused by the periodic change of both forces.

Researchers have conducted wind tunnel experiments on the flow around the square cylinder (Wang et al., 2021; Zhao et al., 2021). However, the wind tunnel experiment is costly and inefficient in obtaining large experimental data. Compared with traditional experiments, numerical simulation and algorithms have the advantages of convenient modeling, low cost, and high computational efficiency for predicting mechanical behavior (Sun et al., 2020a; Luo et al., 2020; Ma et al., 2020). It can also conveniently simulate complex structures’ flow fields and assist engineering problems effectively. Numerical simulation has been widely used in the drag reduction study of various bluff bodies, including square cylinders (Bearman, 1998). Researchers have shown great interest in the flow around square cylinders (Yen and Wu, 2012). The drag reduction control methods for bluff body flow are mainly divided into active and passive control methods. The active control aims to regulate the flow by applying additional external energy. The passive control method changed the shape of the bluff body or added other microstructures. The passive control method is simpler and less expensive (Koide et al., 2006; Kim and Kim, 2012; Bahrami and Hacışevki, 2019).

When the fluid flows through the front edge of the square cylinder, the surface of the square cylinder will hinder the fluid. Also, the high-pressure fluid near the front edge promotes the developing boundary layer to gradually develop to both sides of the square cylinder. Blockage occurs under the influence of viscosity, which loses kinetic energy and causes the fluid to slow down gradually. At the same time, the force generated by the pressure is not enough to surround the entire back of the square cylinder under the high Reynolds number. Still, a separation point is generated near the maximum width of the square cylinder, and the separated fluid forms an unstable shear layer. Since the flow velocity of the outer part near the free flow area is greater than that of the inner part, the fluid tends to rotate and disperse into several vortices. In addition, the strength of the vortex gradually weakens due to the existence of fluid viscosity during the downstream movement of the vortex. After the boundary layer detaches from the surface at the separation point, a wake area is formed at the tail of the square cylinder. Also, the surrounding flow drag is formed under the action of the pressure difference between the front edge and the wake area. When the separation point is further back, the wake area is smaller. Also, the pressure difference resistance acting on the square cylinder is smaller to reduce the drag.

Authors previously reduced drag force by placing cylinders upstream of square cylinders. Earlier, Lesage and Gartshore (1987) placed two-dimensional (2D) plates, circular cylinders, and square cylinders upstream and computed their drag force. Their work has shown that the drag force of superimposed bluff bodies with other cylinders is significantly lower than that of a single bluff body. Igarashi (1997) and Mangal et al. (2014) also use small rods to reduce the drag force of the square cylinder. The authors also modified cylinder surfaces to reduce the drag force to simplify practical applications. He et al. (2014) used particle image velocimetry to study the flow around a square cylinder with front cut corners. The authors found that this structure reduced drag and discussed the fluid dynamical mechanism. Tong et al. (2015) used chamfered corners to reduce the drag of 2D square cylinders with a sharp-angled square and octagonal cross section. The authors found that the chamfered structure reduces drag force compared with sharp corners.

The study also reported microstructures to enhance cylindrical drag reduction. But these solutions are mostly used on the inner wall of the pipes. Costantini et al. (2018) studied the drag reduction caused by the superhydrophobic surface with grooved microstructures in turbulent pipes. Jin et al. (2019) conducted a super large eddy simulation (VLES) on the passive drag reduction of a 2D square cylinder based on OpenFOAM. Lorite-Díez et al. (2020) studied the drag reduction of three-dimensional bluff bodies with different rear cavities under crosswind conditions. When the free flow is aligned with the object, the curved cavity provides stronger wave attenuation and wake bistableness than the straight cavity. Moreover, the drag force of a curved cavity is smaller than that of a straight cavity. Machine learning and deep learning techniques have been widely used in engineering research (Jiang et al., 2021a; Jiang et al., 2021b; Huang et al., 2021; Huang et al., 2022). The neural network is a kind of neural network that simulates the human brain to realize artificial intelligence-like machine learning technology. This study also adopts a deep neural network for resistance prediction (DNN).

Many previous studies have reported the drag reduction of 2D square cylinders, which failed to reflect the drag of real structures. Also, some studies focus on the drag force of the inner wall of the square cylinder modified by the microstructure. Reducing drag force by microstructured the outer surface of the square cylinder is a challenging problem. The study performed the 3D numerical simulation of the flow around the square cylinder based on the numerical simulation. The passive control method is used to achieve drag reduction by adding microstructured patches to the square cylinder. The square cylinder can be divided into convex and concave structures for simulation, including several patch sizes. The optimal drag reduction structure is finally found compared with the smooth square cylinder. Optimizing the surface structure can reduce drag and improve the stability of the structure of the square cylinder. For example, it may provide a reference for the supporting structure of the bridge underwater.

## Materials and Methods

### Calculation Model

The Spalart-Allmaras turbulence model only needs to solve the transport equation. In the Spalart-Allmaras model, the transport variables are the same as the turbulent kinematic viscosity except for the near-wall (viscous effect) region. The transport equation for the modified turbulent viscosity is

$$\frac{\partial }{\partial t}\left(\rho \tilde{v}\right)+\frac{\partial }{\partial x_{i}}\left(\rho \tilde{v}u_{i}\right)=G_{v}+\frac{1}{\sigma_{\tilde{v}}}\left[\frac{\partial }{\partial x_{j}}\left\{\left(\mu +\rho \tilde{v}\right)\frac{\partial \tilde{v}}{\partial x_{j}}\right\}+C_{b2}\rho {\left(\frac{\partial \tilde{v}}{\partial x_{j}}\right)}^{2}\right]-Y_{v}+S_{\tilde{v}},$$

where *G*_*v*_ is the generation of turbulent viscosity, *Y*_*v*_ is the reduction in turbulent viscosity in the near-wall region. $\sigma_{\tilde{v}}$ and *C*_*b2*_ is a constant, ν is the molecular kinematic viscosity. $S_{\tilde{v}}$ is a user-defined source item.

At the wall, the turbulent kinematic viscosity is set to zero. When the calculation grid is fine enough to calculate the laminar bottom layer, the wall shear stress is solved by the laminar flow stress-strain relationship, namely,

$$\frac{u}{u_{\tau }}=\frac{\rho u_{\tau }y}{\mu }.$$

Assuming that the centroid of the mesh adjacent to the wall falls in the logarithmic region of the boundary layer, then according to the wall law,

$$\frac{u}{u_{\tau }}=\frac{1}{k}\ln E\left(\frac{\rho u_{\tau }y}{\mu }\right),k=0.4187,E=9.793,$$

where *u* is the velocity parallel to the wall, *μ*_*τ*_ is the friction velocity, and *y* is the distance from the wall.

(1) The calculation area is 260 mm × 180 mm × 180 mm, the size of the square cylinder is 20 mm × 20 mm × 20 mm, and the square cylinder is located at the center of the calculation area. The distance between the square cylinder model and the velocity inlet and pressure outlet is 120 mm, and the model is 80 mm away from other boundaries.
(2) The fluid parameters are water, density 998.2 kg/m3, viscosity 0.001003 Pa.s, the left side is the water inlet velocity, the inlet velocity is 2 m/s, and the right side is the pressure outlet, the pressure is 0, around. It is a non-slip wall boundary condition.
(3) Re ≈ 40, 000 from the calculation parameters, and the Spalart-Allmaras turbulence model is selected to calculate the flow field. In this case, the model is valid for the wall in the near-wall area. Therefore, the requirements for the quality of the boundary layer grid are relatively high. There is no apparent interface between the fluid inside and outside the boundary layer. In practical applications, it is generally artificially stipulated that the boundary layer thickness is 0.99 times the free velocity from the wall to the flow velocity.

Calculation method of the thickness of the boundary layer of the flat plate:

$$\;\delta \left(L\right)=0.035\frac{L}{Re^{1/7}}∼0.214\mathrm{mm}.$$

According to the thickness of the boundary layer calculated by the above formula, set the boundary layer as follows: Set the boundary layer around the square cylinder, the total thickness of the boundary layer is 10 mm, and there are 20 layers in total, which meet the requirements of the above formula.

Grid setting: the grid size of the square cylinder surface is 0.5 mm, the grid size of other water areas except the boundary layer is 3 mm, and the total number of grids is 1.18 million. Various parameters are shown in Table 1.

**TABLE 1:** Various parameters were used to model the flow past the square cylinder.

Surface pit ratio	b/20	1%	5%	10%	20%	30%	50%
Value	b(mm)	0.2	1	2	4	6	10

### Validation of the Numerical Simulation

**FIGURE 1:**
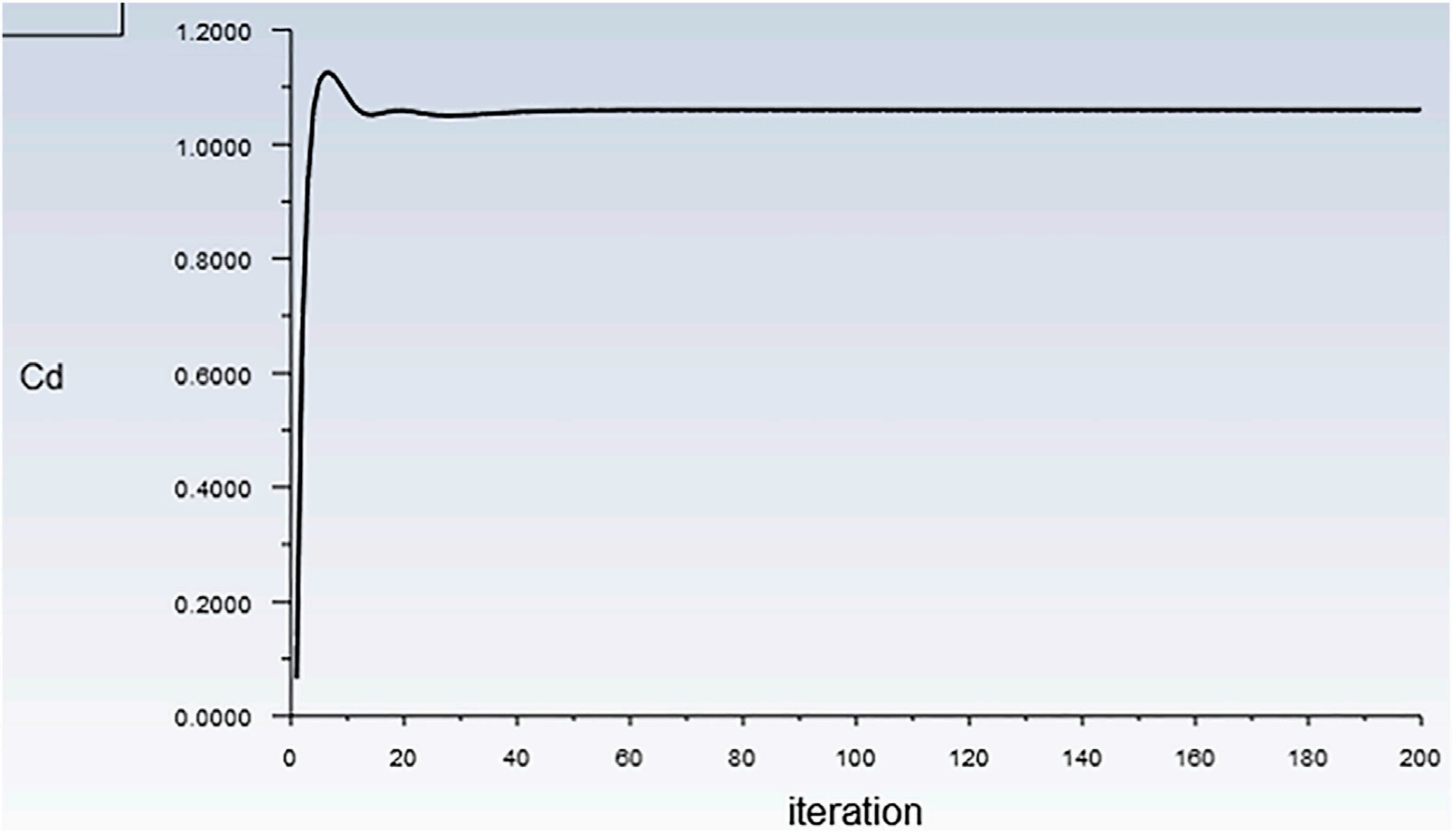
The iterative curve of the drag coefficient.

**FIGURE 2:**
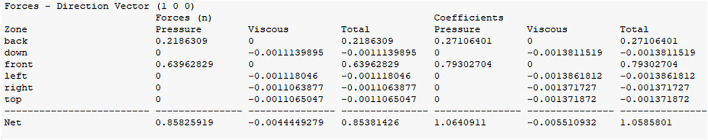
Drag force on all sides.

**FIGURE 3:**
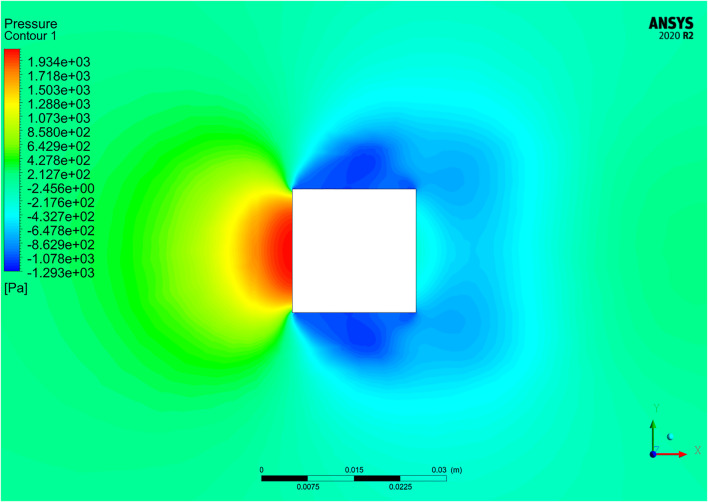
Pressure cloud diagram.

**FIGURE 4:**
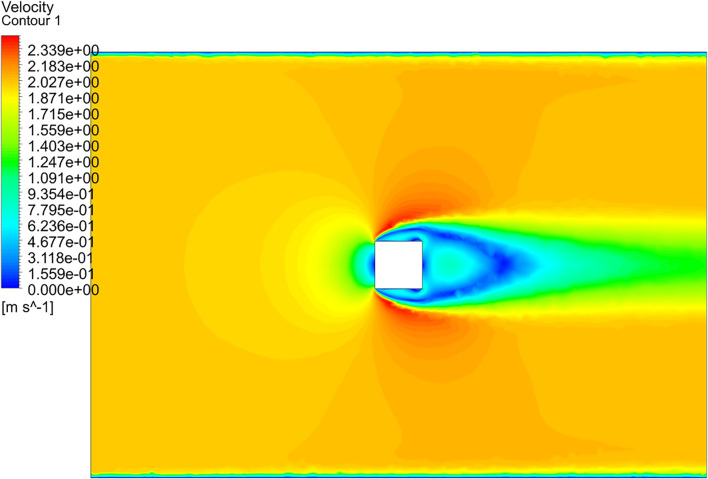
Velocity cloud diagram.

Numerical results from other experiments are compared to validate the mathematical model. The calculated drag coefficient iteration curve is shown in Figure 1. According to the theory of fluid mechanics, the drag forces of an object completely immersed in the fluid are mainly composed of the pressure on the front and rear surfaces of the object and the viscous drag on the side. The pressure drag forces are decisive at higher Reynolds numbers. It can be seen from Figure 2 that the drag force mainly includes the drag force of the pressure difference between the front and rear surfaces, which is consistent with the fluid mechanics' theory of the flow around the body. Figure 3 shows that the pressure on the front surface is the largest, so the pressure drag forces were mainly provided by the front surface (0.6396N). Figure 4 also shows that the velocity gradient on the left and right surfaces changes significantly, and the viscous force mainly comes from this. Thus, both sides mainly provide viscous drag forces. The numerical simulation results show that the pressure on the front and rear surfaces of the object primarily contributes to the drag forces, which also validates that the pressure difference primarily contributes to the drag forces at high Reynolds numbers. Finally, the smooth square cylinder has a drag force of 0.85381 and a drag coefficient of 1.0585. The drag coefficient of the square cylinder obtained from the experiment is 1.05. The drag coefficient obtained by numerical simulation is close to the experiment and the relative error is 6.65%. It shows that the numerical simulation in this study is accurate and reliable.

### Numerical Simulation Conditions

**FIGURE 5:**
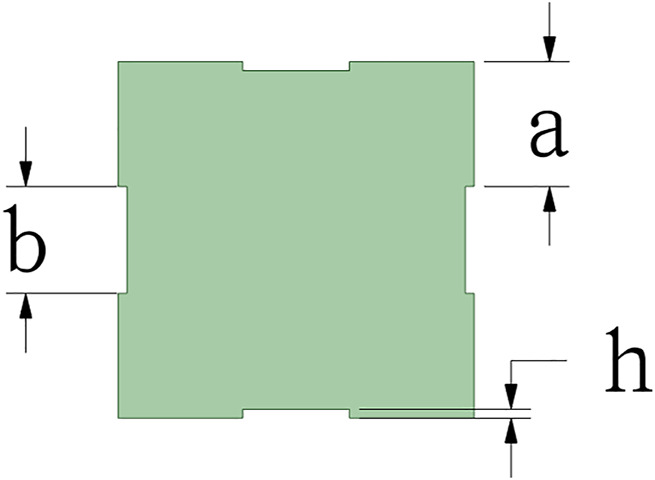
Schematic diagram of square cylinder patch.

We tried to study the influence of different patch sizes on the drag force of the square cylinder. The patch size is shown in Figure 5, and the following equation needs to be satisfied:

$$\sum_{1}^{n+1}a_{i}+b\;*\;n=20,$$

where n is the number of patches, b is the patch length, h is the patch width, and a is the interval length between patches. To ensure the integrity of the square cylinder, h<a must be satisfied. When h takes a positive value, the patch is “sag.” Also, when h takes a negative value, the patch is “raised.” According to the requirements of the above geometric dimensions, the calculation can be divided into the following working conditions (Table 2). The result is measured by the ratio of the surface area occupied by the pits. For its drag reduction effect, some values of the surface pit ratio are shown in Table 3, and other ratios can be interpolated.

**TABLE 2:** Different working conditions.

b (mm)	n	a (mm)	h (mm)
10	1	5	±0.5, ±1, ±2, ±3, ±4
6	1	7	±0.5, ±1, ±2, ±3, ±4, ±5, ±6
	2	3, 2, 3	±0.5, ±1, ±2
	3	0.5	±0.1, ±0.2, ±0.3, ±0.4
4	1	8	±0.5, ±1, ±2, ±3, ±4, ±5, ±6, ±7
	2	4	±0.5, ±1, ±2, ±3
	3	2	±0.5, ±1
2	1	9	±0.5, ±1, ±2, ±3, ±4, ±5, ±6, ±7, ±8
	2	6, 4, 6	±0.5, ±1, ±2, ±3, ±4, ±5
	3	3.5	±0.5, ±1, ±2, ±3
	4	2.4	±0.5, ±1, ±2
1	1	9.5	±0.5, ±1, ±2, ±3, ±4, ±5, ±6, ±7, ±8, ±9
	2	6	±0.5, ±1, ±2, ±3, ±4, ±5
	3	5, 3.5, 3.5, 5	±0.5, ±1, ±2, ±3, ±4
	4	3.2	±0.5, ±1, ±2, ±3
	5	2.5	±0.5, ±1, ±2
	6	2	±0.5, ±1

**TABLE 3:** Values of the surface patch ratio.

Surface patch ratio	b/20	1%	5%	10%	20%	30%	50%
Value	b (mm)	0.2	1	2	4	6	10

### Deep Neural Network Model

The deep neural network (DNN) algorithm was used to build a type prediction model. We use the numerical simulation data to construct a DNN network and randomly divide it into a training and a validation set to construct the prediction model. DNN, also known as a multi-layer perceptual network, is mainly divided into input layer, hidden layer, and output layer. In this study, the conditional data and result data of numerical simulation under multiple sets of working conditions are put into the set network model for training. DNN thoroughly learns the impedance results under different working conditions and assigns the weights of parameters according to the data structure. In this way, DNN can help us predict the resistance effect based on the previously trained model under new working conditions. To guarantee the accuracy of the DNN prediction model, we performed 500 training sessions on the model to find the best parameter combination.

## Results

**FIGURE 6:**
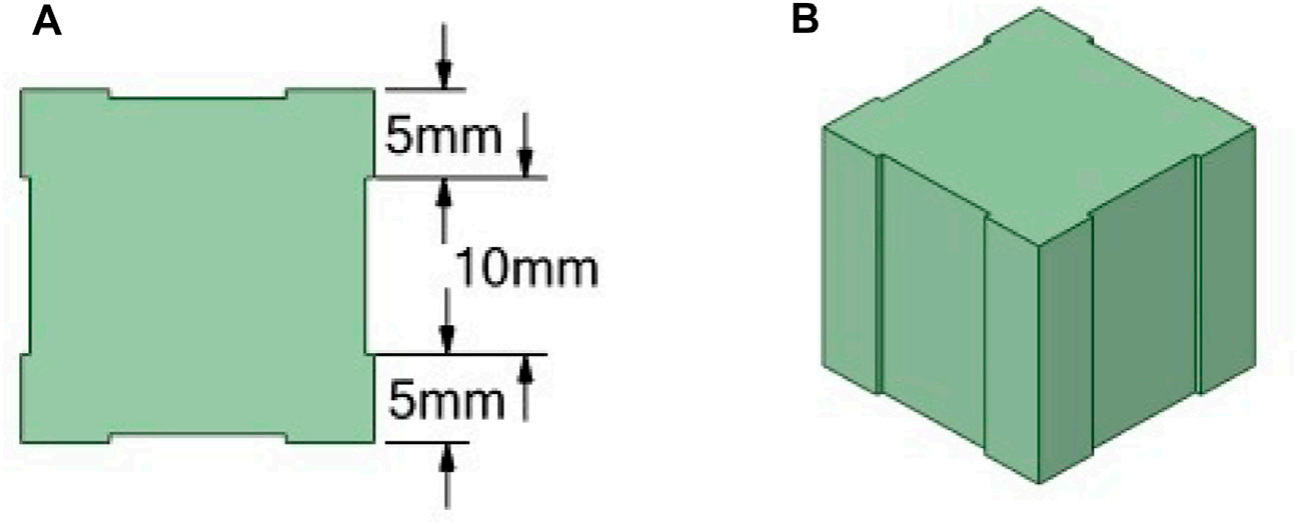
The **(A)** 2D **(B)** 3D patch geometry model of the cylinder (b = 10 mm, a = 5 mm).

**FIGURE 7:**
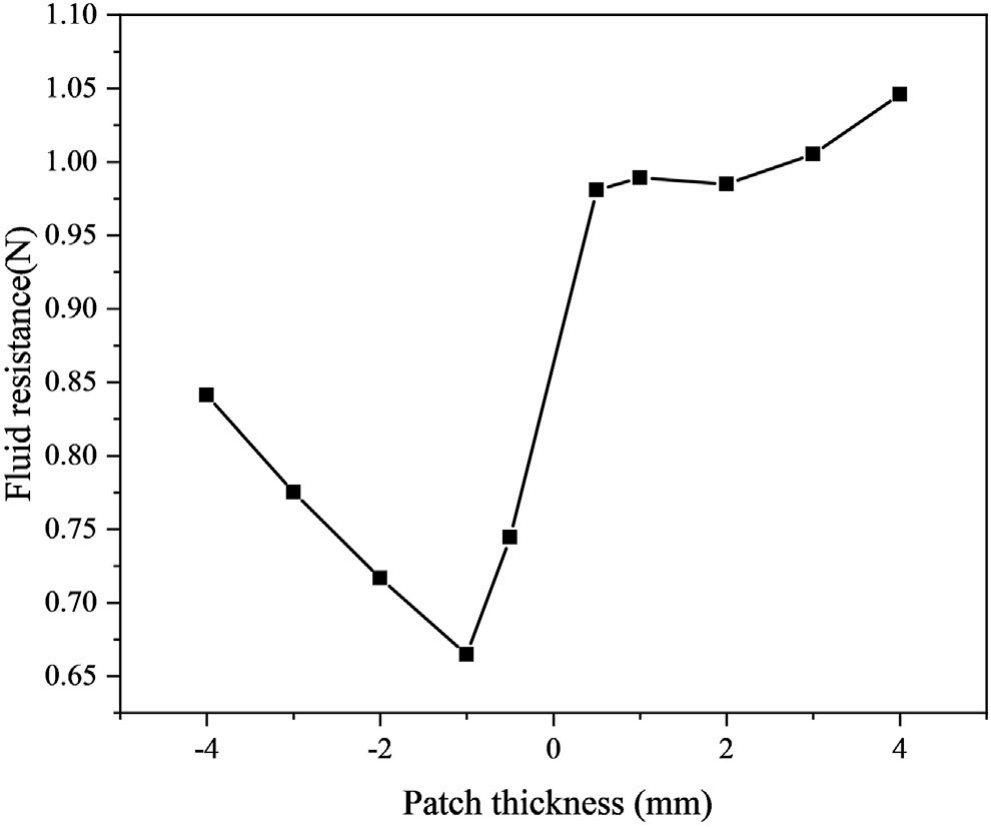
The drag force under different patch thicknesses when b = 10 mm and a = 5 mm.

(1) When b = 10 mm, a = 5 mm, and n = 1. The schematic diagram of the model is shown in Figures 6A,B. The results show that the drag force is the smallest when the patch thickness h = −1 mm (0.66477 N). The surface protrusions account for 50% of the entire area, reducing the drag by 22.1%. Then we changed h to calculate the drag force separately(b = 6 mm, n = 1, 2, 3), as shown in Figure 7. When the thickness is h = -3 mm (b = 6 mm, a = 7 mm, n = 1), the drag force is the smallest, namely 0.74369N. The surface protrusion accounts for 30% of the entire area, reducing the drag by 12.9%.

When b = 6 mm, a = 3 mm, 2 mm, 3 mm, n = 2, h = −2 mm, the drag force is the least, namely 0.70819N. Also, the surface protrusion occupies the entire area with 60%, reducing the drag by 17.1%. When b = 6 mm, a = 0.5 mm, n = 3, h = -0.1 mm the drag force is the smallest, namely 0.70287N. The surface protrusion accounts for the entire area 90%, reducing the drag by 17.7%.

**FIGURE 8:**
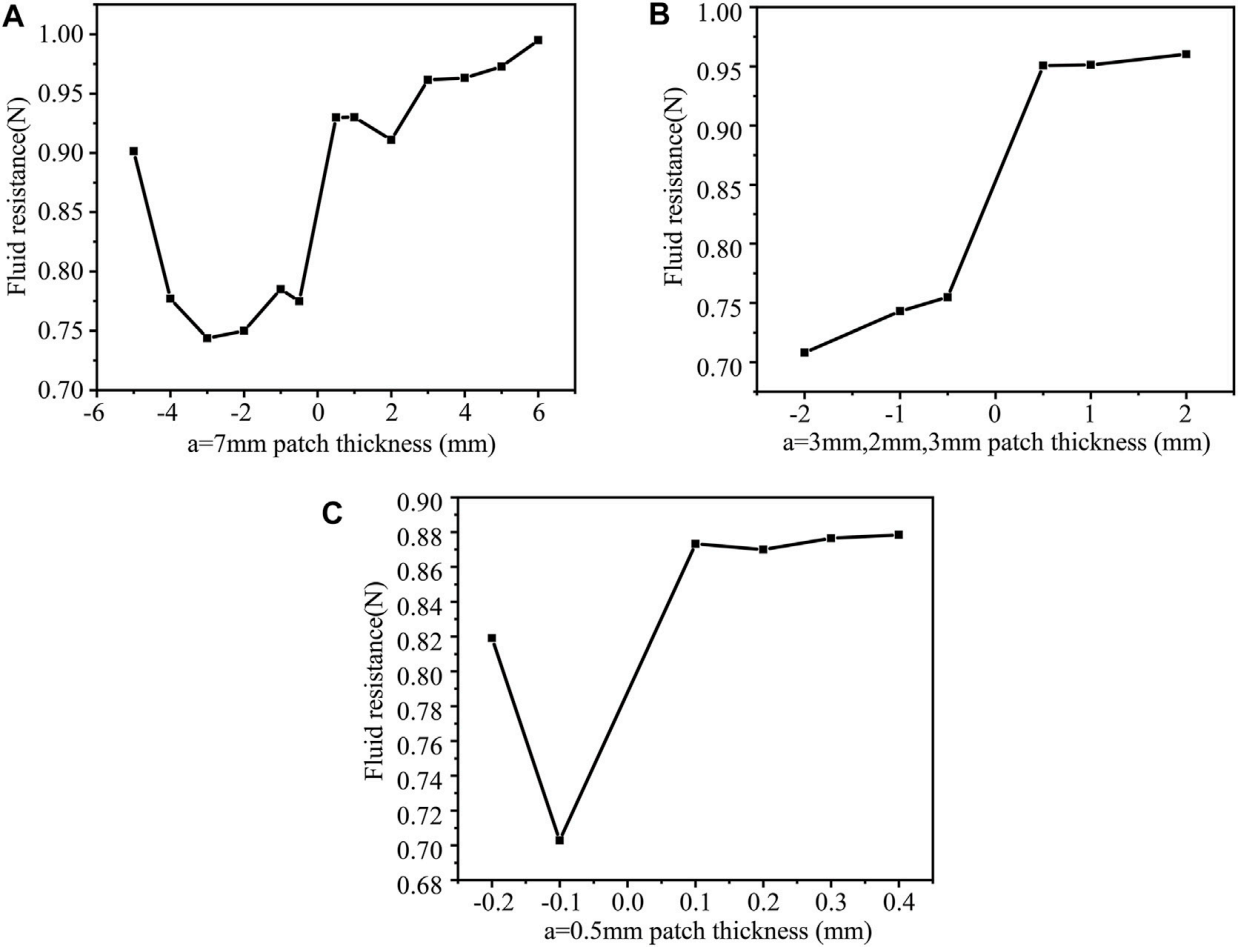
The drag force under different patch numbers and different thickness patches (b = 6 mm). when **(A)** a = 7 mm; **(B)** a = 3mm, 2 mm, 3 mm; **(C)** a = 0.5 mm.

When a = 7 mm, h = −5 mm, the convex structure increased drag force. Figures 8A–C shows that not all concave patch structures have increased drag force, and only some are ineffective in reducing drag force. When the surface protrusions account for 90% of the entire area (n = 3), the square cylinder has the smallest drag force, 0.70287N (drag reduction of 17.7%).

**FIGURE 9:**
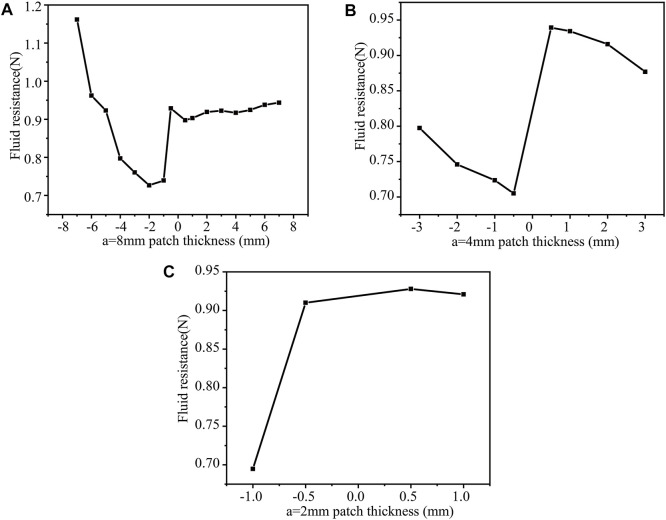
b = 4 mm, the drag force under different patch numbers and different thickness patches **(A)** a = 8 mm; **(B)** a = 4 mm; **(C)** a = 2 mm.

(2) When b = 4 mm, n = 1, 2, 3, then change h to calculate the drag force separately for a = 8 mm (Figure 9A), a = 4 mm (Figure 9B), a = 2 mm (Figure 9C). When the thickness h = −2 mm (b = 4 mm, a = 8 mm, n = 1), the drag force is the least, 0.72654N. The surface protrusions account for 20% of the entire area, reducing the drag by 14.9%. In this case, when the patch size is relatively large, the drag force increase is significant. Consequently, reasonable patch size is essential.

When the patch thickness h = −0.5 mm (b = 4 mm, a = 4 mm, n = 2), the drag force is 0.70498N. The percentage of the surface protrusion to the entire area is 40%, reducing the drag by 17.4%. The concave patch structure also shows increased drag force. The drag force is the smallest (0.69471N) when h = −1 mm(b = 4 mm, a = 2 mm, n = 3). The surface protrusion accounts for 60% of the entire area, reducing the drag by 18.6%.

Taken together, when the surface protrusions account for 40% of the entire area (n = 2), the square cylinder has the least drag force, 0.69471N, reducing the drag by 18.6%.

**FIGURE 10:**
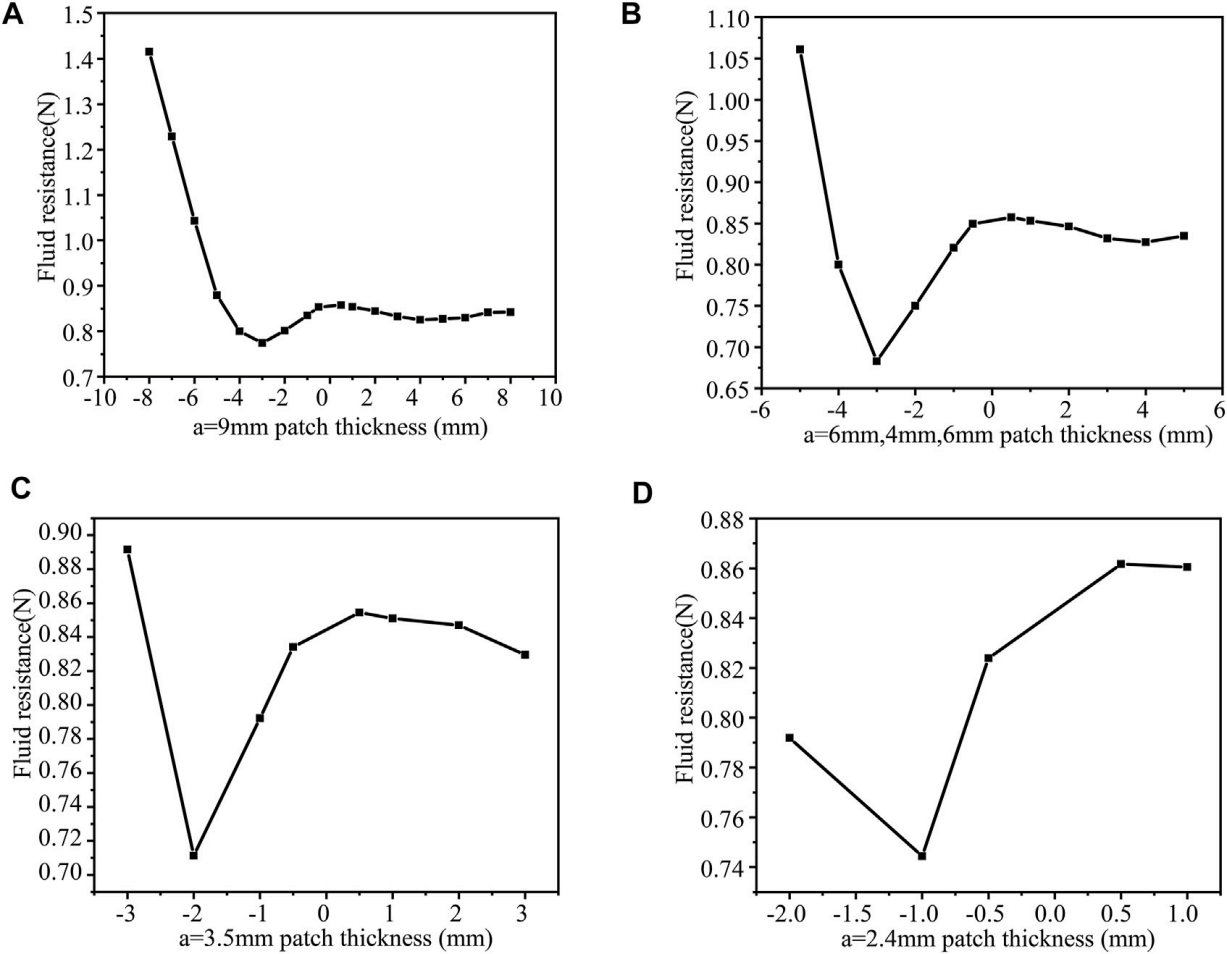
b = 2 mm, the drag force under different patch numbers and different thickness patches in the case of **(A)** a = 9 mm **(B)** a = 6 mm, 4 mm, 6 mm **(C)** a = 3.5 mm **(D)** 2.4 mm.

(3) When b = 2 mm, n = 1, 2, 3, 4, then change h and calculate the drag force separately a = 9 mm (Figure 10A), a = 6 mm (Figure 10B), a = 3.5 mm(Figure 10C), a = 2.4 mm (Figure 10D). When the thickness h = −3mm, the drag force is the least, 0.77414N (b = 2 mm, a = 9 mm, n = 1). The surface protrusions account for 10% of the entire area, reducing the drag by 9.3%. The situation is the same as before. As a result, drag force is significantly increased when the protrusion size is relatively large. The drag force is the smallest (0.68278N) when h = −3 mm, and the drag force (b = 2 mm, a = 6 mm, 4 mm, 6 mm, n = 2). The surface protrusion occupies 20% of the entire area, reducing the drag by 20%. The drag force is the smallest (0.71127N) when the patch thickness h = −2 mm (b = 2 mm, a = 3.5 mm, n = 3). The surface protrusion accounts for 30% of the entire area, reducing the drag by 16.7%. The drag force is the smallest 0.74444N when h = −1 mm (b = 2mm, a = 2.4mm, n = 4). The surface protrusion accounts for 40% of the entire area, reducing the drag by 12.8%.

When the surface protrusion accounts for 20% of the entire area (n = 2), the square cylinder has the smallest drag force, 0.68278N, reducing the drag by 20%.

**FIGURE 11:**
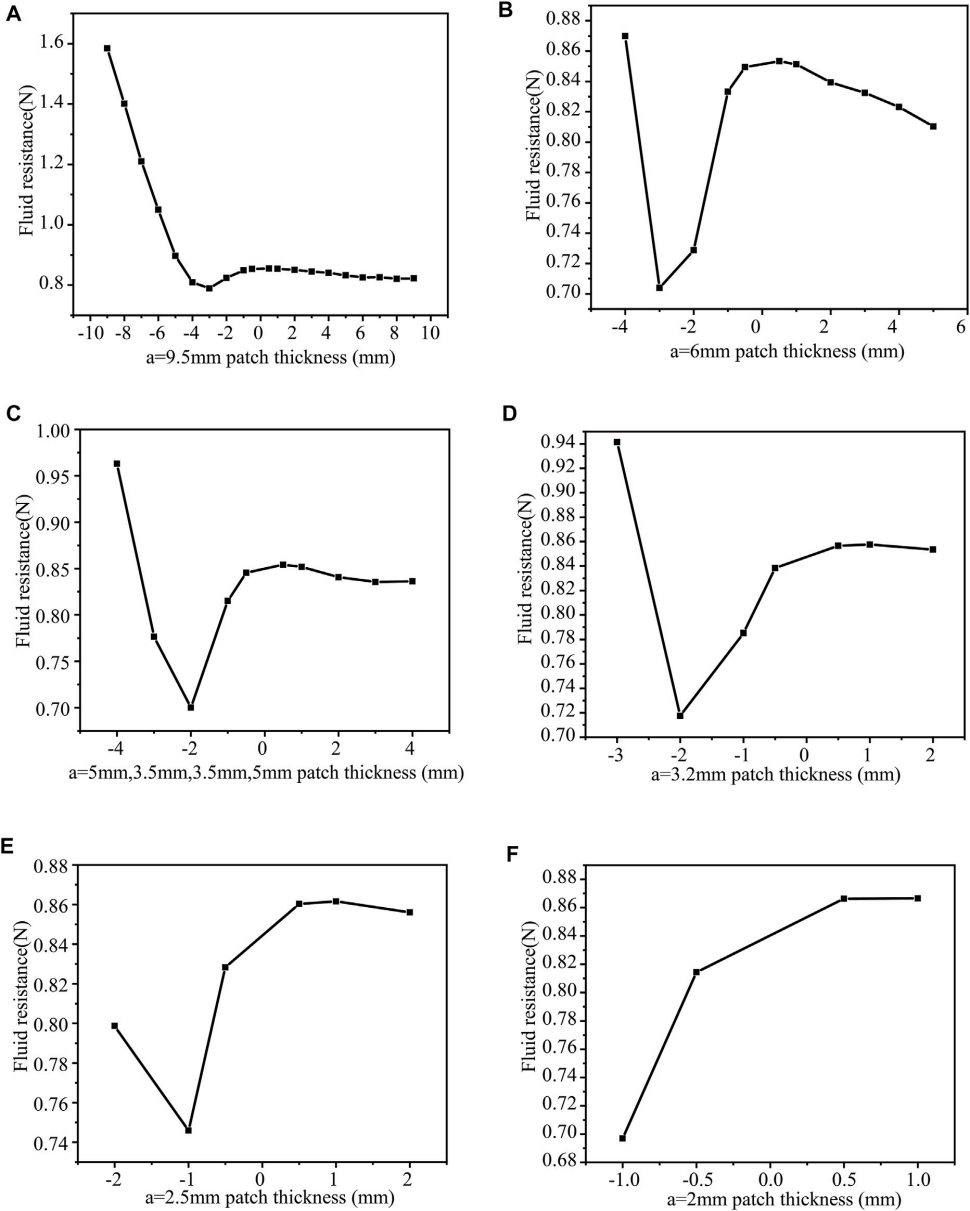
b = 1 mm, the number of patches and the drag force under different thickness patches **(A)** a = 9.5 mm; **(B)** a = 6 mm **(C)** a = 5 mm **(D)** a = 3.2 mm **(E)** a = 2.5 mm **(F)** a = 2 mm.

(5) When b = 1 mm, n = 1, 2, 3, 4, 5, 6, change h and calculate the drag force separately a = 9.5 mm (Figure 11A), a = 6 mm (Figure 11B), a = 3.5 mm (Figure 11C), a = 2.4 mm (Figure 11D), a = 2.5 mm (Figure 11E), a = 2 mm (Figure 11F). When h = −3mm, the drag force is the least, 0.78932N (b = 1 mm, a = 9.5 mm, n = 1). The surface protrusions account for 5% of the entire area, reducing the drag by 7.6%. The drag force is the smallest (0.70387N) when the patch thickness is h = −3 mm (b = 1 mm, a = 6 mm, n = 2). The surface protrusion accounts for 10% of the entire area, reducing the drag by 17.6%. The drag force is the smallest 0.70015N when the patch thickness h = −2 mm (b = 1 mm, a = 5 mm, 3.5 mm, 5 mm, n = 3). Therefore, surface protrusion accounts for 15% of the entire area, reducing the drag by 18%.

The drag force is the smallest 0.71761N when the patch thickness is h = −2 mm (b = 1 mm, a = 3.2 mm, n = 4). Surface protrusion accounts for 20% of the entire area, reducing the drag by 15.9%. The drag force is the smallest 0.74585N when the patch thickness h = −1 mm (b = 1 mm, a = 2.5 mm, n = 5). Surface protrusion accounts for 25% of the entire area, reducing the drag by 12.6%. The drag force is the smallest (0.69693N) when the thickness of the patch is h = −1 mm (b = 1 mm, a = 2 mm, n = 6). The surface protrusion accounts for 30% of the entire area, reducing the drag by 18.4%.

On the whole, when the surface protrusion accounts for 30% of the entire area (n = 6), the square cylinder has the least drag force, 0.69693N. At this time, reducing the drag by 18.4%.

**FIGURE 12:**
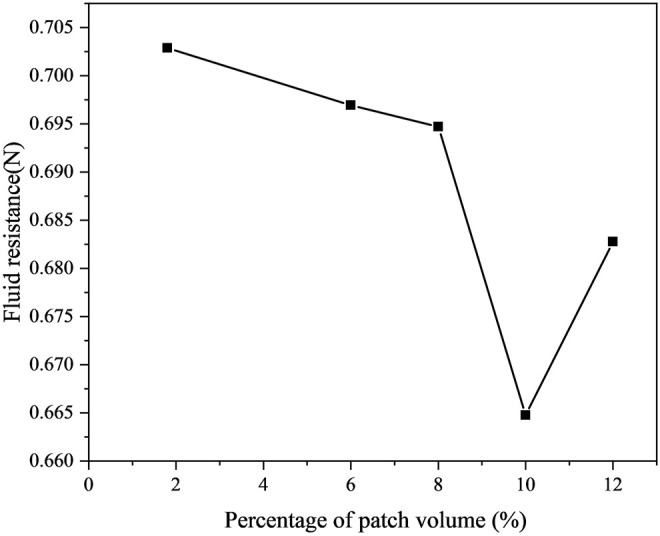
Relationship between volume percentage and minimum drag force.

**FIGURE 13:**
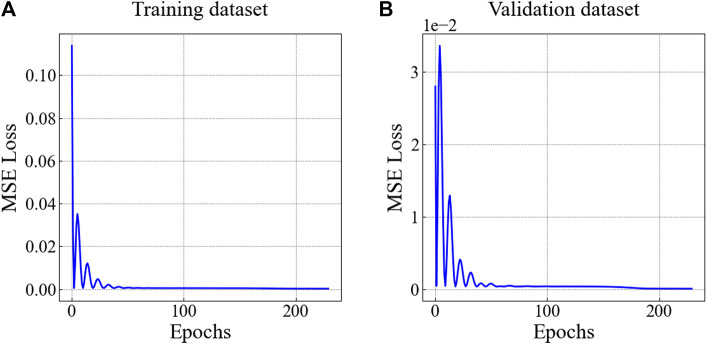
**(A)** MSE loss based on a training set of the DNN model **(B)** MSE loss based on a validation set of the DNN model.

Select the optimal drag reduction structure for each working condition, as shown in Table 4. The volume percentage and the minimum drag force was shown in Figure 12. At the same time, we performed 500 training sessions on the model to find the optimal parameter combination to ensure the accuracy of the DNN prediction model. Figure 13A shows the DNN model’s training effect based on the drag force data of the numerical simulation. The results indicate that the DNN model based on numerical simulation data has an excellent prediction for drag reduction. Moreover, Figure 13B demonstrates the robust performance of trained mature DNN models using the validation dataset. We found that the DNN prediction model has performance, as confirmed by the validation dataset, demonstrating the robustness of the numerical simulation data.

**TABLE 4:** The optimal drag reduction structure for each working condition.

No.	Patch length	Number of patches	Patch thickness (convex)	Volume percentage (%)	Minimum drag force (N)
1	1	6	1	6	0.69693
2	2	2	3	12	0.68278
3	4	2	1	8	0.69471
4	6	3	0.1	1.8	0.70287
5	10	1	1	10	0.66477

## Discussion

Numerical simulation shows that the drag reduction performance of the convex square cylinder is better than that of the concave ones. However, not all raised structures reduced the drag. The drag reduction performance mainly depends on the degree of its protrusion. Too large a protrusion will increase the drag force. However, the drag reduction effect of the concave patch structure is not apparent, and the drag is increased in many cases.

When the fluid particles flow through the front edge of the square cylinder, the surface of the square cylinder will hinder the fluid, and the high-pressure fluid near the front edge promotes the developing boundary layer to gradually develop to both sides of the square cylinder. Blockage occurs under the influence of viscosity, which loses kinetic energy and causes the fluid particles to slow down gradually. At the same time, under the condition of a high Reynolds number, the force generated by the pressure is not enough to surround the entire back of the square cylinder. Still, a separation point is generated near the maximum width of the square cylinder section, and the separated fluid forms an unstable shear layer. Since the flow velocity of the outer part near the free flow area is greater than that of the inner part, the fluid tends to rotate and disperse into several vortices. In addition, during the downstream movement of the vortex, the strength of the vortex gradually weakens due to the existence of fluid viscosity. After the boundary layer detaches from the surface of the square cylinder at the separation point, a wake area is formed at the tail of the square cylinder, and the surrounding flow resistance is formed under the action of the pressure difference between the front edge of the square cylinder and the wake area. When the separation point is further back, the wake area is smaller, and the pressure difference resistance acting on the square cylinder is smaller to reduce resistance.

The protrusion patch has a noticeable drag reduction, whereas the pit patch increases the drag. Similar results have been reported in other kinds of literature. Ueda et al. (2009) used numerical and experimental methods to visualize the flow around a square cylinder with a chamfered front edge to explore its drag reduction mechanism. Although the square-pillar structure of the literature is different from that of this study, they are all convex structures, which also achieve a better drag reduction effect. Kurata et al. (2009) studied the drag reduction of a right-angled cylinder with a leading-edge chamfer and an aspect ratio of less than or equal to the unit, which is also a convex structure and has a better drag reduction effect. However, they only explored the drag reduction of a convex side without considering the pits. The study further changed the structure of the four directions of the square cylinder. Thus, the work made a supplement to the research of others. The structure of the square cylinder in this article also has a better drag reduction performance.

Song et al. (2019) studied the drag reduction of the grooved cylinder, and the results showed that under the supercritical Reynolds number, the drag reduction is significant under the appropriate groove structure parameters. The results show that the drag force reduction of the pit-patched square cylinder is not significant. The limited selection of pit sizes may increase the drag force. This is also a supplement to the work of others.

We use the passive control method to study square cylinder drag reduction, which is similar to Jin et al. For drag reduction, the passive control method is usually used to cut corners at the front edge of a square cylinder. First, Jin adopts the previously studied control method (called CM-1, or control method 1) and conducts numerical research. On this basis, a new control method was proposed. That is, an additional cut corner (CM-2, control method 2) was used to test the performance of the drag reduction. Both control methods of CM-1 and CM-2 provide significant drag reduction. The drag reduction is as high as 48% and 61%, respectively, compared with the smooth square cylinder. In addition, the newly proposed control method is more effective than the previous control method (Jin et al., 2019).

Numerous researchers have previously investigated the drag reduction of hollow cylinders. Durhasan (2020) studied the drag reduction of hollow cylinders with slits. He observed all the gap ratio structures compared with solid cylinders. The slit cylinders have reduced the drag coefficient by 42%. Wei et al. (2013) used computational fluid dynamics (CFD) methods to analyze the drag reduction ability of triangular grooves on the flow in the pipe. The study found that the triangular groove structure weakened the velocity fluctuation and intensity of the turbulent burst of the boundary layer and macroscopically reduced the velocity gradient in the near-wall area, thereby reducing the surface frictional drag force, with a maximum drag reduction rate of 6.93%. We applied the same numerical simulation method. The cylindrical pipe they studied and our square cylinder can be used as an analogy reference. Yanuar et al. (2017) studied the influence of agar jelly coating on the flow drag force reduction in rectangular pipes, and the results showed that the maximum drag force reduction was about 19%. They also use a passive control method to study the drag reduction mechanism through additive polymers.

The splitter plate may achieve a good performance of drag reduction of the square cylinders. Dash et al. (2020) carried out a numerical study on the drag force reduction of the square cylinder using the double splitter plate. The study showed that the double splitter plate suppressed the shedding of the von Karman vortex and lift fluctuations and produced good drag reduction (≈21%) when less than half the length of the single splitter plate. However, Ma et al. (2018) applied comprehensive methods to promote drag reduction performance further. They numerically simulate the drag reduction in a structure with round rods arranged upstream of the square cylinder and a splitter plate downstream. The study shows that the average drag coefficient of the maximum percentage reduction is 68.76%. Although the passive control methods adopted by these researchers are different, they have achieved different levels of drag reduction. Data fusion technology and intelligent algorithms may help flow forecasting (Sun et al., 2020b; Sun et al., 2020c; Tan et al., 2020). The DNN method in this study also showed excellent prediction performance.

## Conclusion

Under the typical high Reynolds number Re = 40,000, this study uses the Spalart-Allmaras turbulence model to calculate the flow field. Based on verifying the accuracy of the numerical results, the effect of different patch sizes on the drag force of the square cylinder is studied. The convex square cylinder of the patch has a better drag reduction effect than the concave patch. Concave patches do not significantly reduce drag and sometimes even increase drag. The drag reduction effect of the convex structure of the square cylinder is better than that of the concave structure. Too large a convexity will increase the drag force of the structure. When the convex area of the patch accounts for 10% of the total area of the square cylinder (b = 10 mm, n = 1, h = 1 mm), the drag reduction performance is the best (22.1%). Also, the DNN prediction model demonstrated the robustness of the numerical simulation data.

## Data Availability

The raw data supporting the conclusion of this article will be made available by the authors, without undue reservation.
